# GC-MS Based Metabolite Profiling, Antioxidant and Antimicrobial Properties of Different Solvent Extracts of Malaysian* Plectranthus amboinicus* Leaves

**DOI:** 10.1155/2017/1517683

**Published:** 2017-03-23

**Authors:** Mallappa Kumara Swamy, Greetha Arumugam, Ravinder Kaur, Ali Ghasemzadeh, Mazina Mohd. Yusoff, Uma Rani Sinniah

**Affiliations:** ^1^Department of Crop Science, Faculty of Agriculture, Universiti Putra Malaysia, 43400 Serdang, Selangor, Malaysia; ^2^Laboratory of Natural Products, Institute of Bioscience, Universiti Putra Malaysia, 43400 Serdang, Selangor, Malaysia

## Abstract

This study evaluates the phytochemistry, antioxidant, and antimicrobial effects of* Plectranthus amboinicus* leaves extracted in different solvents. The methanol extract contained the highest total phenolic (94.37 ± 1.24 mg GAE/g) and flavonoid contents (26.90 ± 1.35 mg RE/g) and exhibited the highest DPPH scavenging activity (90.13 ± 3.32%) followed by the acetone extract (80.23 ± 3.26%) at 500 *μ*g/mL concentration. Similarly, the highest ferric ion reduction potential (849.63 ± 30.95 *μ*M of Fe (II)/g dry weight) was exhibited by the methanol extract followed by the acetone extract (695.92 ± 25.44 *μ*M of Fe (II)/g dry weight). The methanol extract showed greater antimicrobial activity against all the tested pathogens (*Bacillus subtilis*, Methicillin-resistant* Staphylococcus aureus*,* Pseudomonas aeruginosa*,* Escherichia coli*, and* Candida albicans*). However, both hexane and acetone extracts failed to inhibit* E. coli*.* S. aureus* and* C. albicans* were more susceptible to all the extracts. Further, GC-MS analysis confirmed the occurrence of a total 46 phytocompounds in different solvent extracts. Some of the major compounds included carvacrol (37.7%), tetracontane (16.6%), squalene (15.6%), tetrapentacontane (13.7%), and Phytol (12.9%). In conclusion, extraction solvents influenced the recovery of phytocompounds and the highest pharmacological activities of the methanol extract could be correlated to the presence of additional bioactive compounds.

## 1. Introduction

In nature, plants encompass a wide range of therapeutically valued bioactive compounds. These natural compounds are widely used in both traditional and modern therapies for improving human health with relatively less or no side effects. Globally, various medicinal plants have been well explored to discover novel drug molecules to combat the threat of ever-increasing human diseases [1–3]. In the human body, cellular mechanisms lead to the generation of unstable compounds such as reactive oxygen species and free radicals, which can destroy living cells and may cause several clinical diseases. A range of synthetic and natural antioxidants are proved to be effective in controlling the activities of free radicals [4]. However, synthetic antioxidants cause adverse effects on the human body. Currently, though natural drugs are developed against human pathogens, the occurrence of drug resistance in microbes is posing a major challenge to the scientific community. Therefore, there is a need to discover novel drugs from the natural sources to benefit mankind.* Plectranthus amboinicus* (Lour.) Spreng. is a medicinal herb (Lamiaceae) which grows naturally in the subtropics and tropics of Africa, Asia, and Australia. The occurrence of about 30 nonvolatile and 76 volatile compounds attributes to its pharmacological properties such as anti-inflammatory, antimicrobial, wound healing, analgesic, antitumor, antiepileptic, antioxidant, and larvicidal actions [2]. Recent studies have suggested the success of this herb against oral, respiratory, skin, cardiovascular, digestive, and urinary diseases [2]. Previous studies have witnessed the existence of significant variations in secondary metabolites accumulation among the same plant species grown under diverse ecological conditions [5, 6]. In general, various solvents are used for extracting plant metabolites from their different parts. Moreover, the extraction of phytocompounds and yield mainly depends on the type of solvents and the method of extraction [7]. Literature survey shows less information on phytochemicals and bioactivities of* P. amboinicus* plants growing under tropical conditions of Malaysia [8]. With this background, we investigated the influence of different solvents to recover higher phytochemicals from a local* P. amboinicus *plant leaves and assessed their polyphenols and antioxidant and antimicrobial activities. Further, GC-MS analysis was carried out to explain the occurrence of bioactive nonvolatile compounds in different solvent extracts.

## 2. Materials and Methods

### 2.1. Plant Material Collection

The wild growing* P. amboinicus* plant material was collected from the forest area near Universiti Putra Malaysia (UPM), Serdang, Selangor, Malaysia, during the month of August 2015. The plant was identified and authenticated taxonomically at the Department of Crop Science, UPM, Malaysia. The voucher specimen, PA-082015, was deposited in the department. The detached leaves from the collected plant materials were washed carefully under running tap water and dried for 1 week at room temperature in a shaded area. The dried leaves were finely powdered by using an electric blender and stored in a plastic bag container for further use at room temperature.

### 2.2. Preparation of Extracts

About 25 grams of powdered leaves of* P. amboinicus* were kept in a beaker to which 100 mL of various organic solvents (methanol, acetone, and hexane) was added and thoroughly shaken. Later, the mixture was placed at room temperature for 48 hrs and stirred 2-3 times a day. After filtering the mixture, the filtrate was evaporated to dryness using rota-vapor. The final extracts were weighed to determine the yield (%) and the dried extracts were stored at 4°C in a refrigerator for further studies.

### 2.3. Determination of Total Phenolic Content in* P. amboinicus* Extracts

Using the FC (Folin-Ciocalteu) colorimetric assay method, total phenolic content occurring in different solvent extracts of* P. amboinicus *leaves was evaluated [4]. Briefly, dried plant extract (0.1 g) was dissolved in 1 mL of distilled water. About 0.1 mL of this solution was later added to a solution containing 20% sodium carbonate solution (1 mL) and 50% FC reagent (0.5 mL). This solution was mixed thoroughly and allowed to stand by at room temperature for 20 min to facilitate the reaction to occur. Afterwards, the absorbance of the solution was recorded at a wavelength of 730 nm against the blank (water). Likewise, a standard calibration curve was generated by using gallic acid (standard) at different concentrations. The gallic acid curve was used to convert the absorbance of different plant extracts into total phenolic content and expressed in mg GAE (gallic acid equivalents) per gram of dried solvent extract.

### 2.4. Determination of Total Flavonoid Content in* P. amboinicus* Extracts

The presence of total flavonoids content in various solvent extracts of* P. amboinicus *leaves was determined by using the colorimeter assay method as described by Swamy et al. [4]. In short, about 0.5 mL of the solvent extract (1 mg/mL) was suspended in 2 mL of distilled water and then added with 150 *μ*L of 5% NaNO_2_ solution and incubated at room temperature for 5 min. Thereafter, the solution was added to 2 mL of NaOH (4%) and 600 *μ*L of AlCl_3_ (10%) and made up to 5 mL using distilled water. The solution was mixed thoroughly and incubated for 15 min at room temperature. The absorbance of the solution was documented at a wavelength of 510 nm against the blank (water). In a similar way, a standard calibration curve was prepared by using the standard, rutin, at different concentrations. By using the standard curve of rutin, total flavonoid content was determined and expressed in mg of RE (rutin equivalents) per gram of dried solvent extract.

### 2.5. In Vitro Antioxidant Activity Evaluation of* P. amboinicus* Extracts

#### 2.5.1. DPPH Free Radical Scavenging Assay

1,1-Diphenyl-2-picrylhydrazyl (DPPH) free radical scavenging assay was used to evaluate the antioxidant activity of different solvent extracts of* P. amboinicus* leaves as explained by Mohanty et al. [9] with minor modifications. In brief, different plant extract in the same volume (0.3 mL) at different concentrations ranging from 100 to 1000 *μ*g/mL was added to 2 mL of DPPH solution (0.1 mM) and placed in dark condition at room temperature for 30 min. The absorbance of this solution was measured at a wavelength of 517 nm against the blank (methanol) by using UV-visible spectrophotometer. Ascorbic acid was used as the standard control. The percentage of inhibiting free radicals by each extracts was calculated by using the equation given below:

(1) Inhibition  activity  %=Absorbance  (control)−Absorbance  (extract)Absorbance  (control)×100.

#### 2.5.2. FRAP (Ferric Reducing Antioxidant Potential) Assay

FRAP assay was used to determine the total antioxidant activity of different solvent extracts of* P. amboinicus* leaves. Briefly, FRAP reagent was freshly prepared by mixing 2.5 mL of FeCl_3_ (20 mmol/L), 2.5 mL of 10 mmol/L TPTZ (2,4,6-tripyridyl-S-triazine), and 25 mL of 0.3 mol/L acetate buffer (pH, 3.6) and incubated at 37°C under dark condition for 20 min. Different solvent extracts (2.0 mL) were added into 2.0 mL of FRAP reagent and the final volume was made up to 10 mL. The mixture was incubated at 25°C for 30 min under dark condition. The absorbance of the solution was recorded at 593 nm using acetate buffer as the blank. Results are expressed in *μ*M Fe (II)/g dry weight and equated with the standard, *α*-tocopherol.

### 2.6. Determination of Antibacterial Activity

The test included a total of four bacterial species, namely,* Bacillus subtilis* B29, Methicillin-resistant* Staphylococcus aureus* (MRSA) (ATCC700698) (gram-positive),* Pseudomonas aeruginosa *(ATCC 15442),* Escherichia coli *E266 (gram-negative), and one fungal species,* Candida albicans* 90028. All microbial strains were procured from the Laboratory of Molecular Biomedicine, Institute of Bioscience, UPM, Serdang, Malaysia. The pure cultures of bacterial strains were subcultured onto Müller-Hinton Agar (MHA) while* C. albicans *9002 was cultured on potato dextrose agar (PDA). The antibacterial properties of different solvent extracts of* P. amboinicus* leaves were evaluated by using disc diffusion method as described by Kumara et al. [10] with little modifications. In short, 10 mg of each solvent extract was dissolved in 1 mL of dimethyl sulfoxide (DMSO) and about 10, 20, and 30 *μ*L of this solution were impregnated on 6 mm sized sterilized filter paper discs. Later, the air-dried discs were placed on individual MHA or PDA plates and previously preinoculated uniformly with a known bacterial or fungal strain, respectively. The discs saturated with DMSO (20 *μ*L) served as a negative control for all microbes while streptomycin (100 mg/mL) and nystatin (100 mg/mL) served as a positive control for bacterial and fungal species, respectively. All microbes were incubated in an incubator at 37°C for 24 hrs to observe the zone of growth inhibition (mm) around each disc. For each microbial strain, the experiment was repeated 3 times.

### 2.7. GC-MS Analysis

Each solvent extract was subjected to GC-MS analysis using the model instrument, GCMS-QP2010 Ultra (Shimadzu Co., Japan) attached with a capillary column DB-1 (0.25 *μ*m film × 0.25 mm I. d. × 30 m length). Analysis was performed by injecting 1 *μ*L of the sample with a split ratio of 20 : 1. Helium gas (99.9%) was used as the carrier gas at a flow rate of 1 mL/min. The analysis was performed in the EI (electron impact) mode with 70 eV of ionization energy. The injector temperature was maintained at 250°C (constant). The column oven temperature was set at 50°C (held for 3 min), raised at 10°C per min to 280°C (held for 3 min), and finally held at 300°C for 10 min. The compounds were identified after comparing the spectral configurations obtained with that of available mass spectral database (NIST and WILEY libraries).

### 2.8. Statistical Analysis

In each experiment, the data recorded was from 3 replications (*n* = 3) and all the results are represented as mean ± SD. One-way analysis of variance (ANOVA) was carried out to compare the data. Further, to determine the statistically significant differences, Tukey's test was performed at *p* < 0.05 level using GraphPad Prism (version 5.0) statistical software.

## 3. Results

The dry weight and final yield of the leaf extract were significantly affected by different solvents used for the extraction (Table 1). The methanol solvent recorded the highest weight of the extract (131.6 ± 2.6 mg) and its yield (1.3%). The extractable elements recovered from different solvents are in the following order, that is, methanol > hexane > acetone. Further, solvent extracts had a significant difference (*p* < 0.05) in their total phenolic and total flavonoid content (Table 2). In the methanol leaf extract, the highest total phenolic content (94.37 ± 1.24 mg GAE/g) and flavonoid content (26.9 ± 1.3 mg RE/g) were recorded. In hexane extract, total phenolic content was found to be 75.39 ± 1.07 mg GAE/g. However, total flavonoid content (16.46 ± 0.9 mg RE/g) was relatively lesser when compared to the acetone extract (21.27 ± 0.8 mg RE/g).

**Figure 1:**
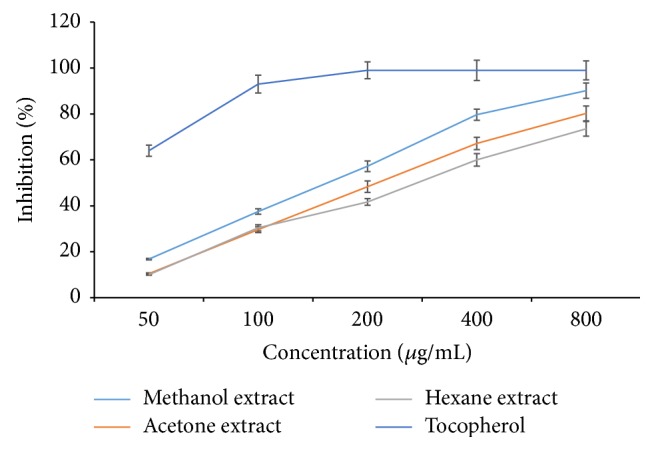
DPPH free radical scavenging activities of various solvent extracts of* P. amboinicus*.

DPPH free radical scavenging property was observed to be dependent on the concentration for all the tested solvent extracts. The methanol extract exhibited the highest DPPH scavenging activity (90.13 ± 3.32%) followed by acetone extract (80.23 ± 3.26%) and hexane extract (73.52 ± 3.18%) at 500 *μ*g/mL concentration (Figure 1).

**Figure 2:**
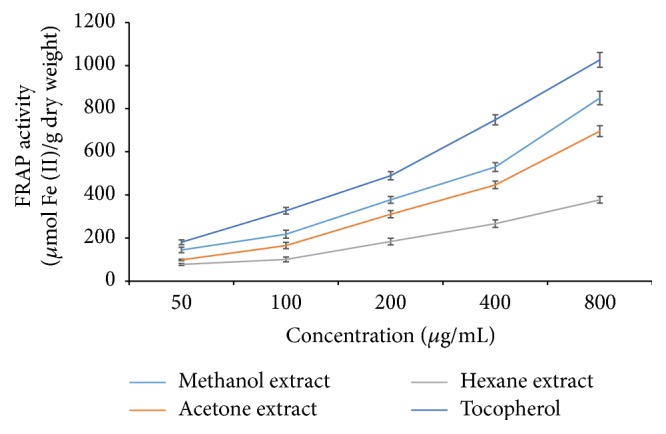
FRAP assay of various solvent extracts of* P. amboinicus*.

However, DPPH scavenging activity of all extracts was inferior to the standard (ascorbic acid). Similar pattern of antioxidant activity was evidenced from FRAP assay method where the highest ferric ion reduction potential was exhibited by the methanol extract (849.63 ± 30.95 *μ*M of Fe (II)/g dry weight) followed by acetone extract with 695.92 ± 25.44 *μ*M of Fe (II)/g dry weight (Figure 2). The lowest FRAP activity was observed in hexane extract (376.98 ± 15.42 *μ*M of Fe (II)/g dry weight). However, the superior activity was evidenced in the standard, *α*-tocopherol.

The microbial inhibitory potential of different solvent extracts of* P*.* amboinicus* is depicted in Table 3. The results clearly showed the existence of a varied and selective antimicrobial activity of different solvent extracts against each microbial species tested. Further, increased concentration of extracts evidenced relatively a higher activity in all the tested microbes irrespective of the solvent. Among all the tested microbes,* S. aureus* (MRSA) and* C. albicans* were more susceptible to all the extracts evaluated. The highest activity was observed in the methanol extract for* B. subtilis* (10.2 ± 0.5 mm),* S. aureus* (MRSA) (09.2 ± 0.3 mm),* P. aeruginosa* ATCC 15442 (07.2 ± 0.2 mm),* E. coli* E266 (08.7 ± 0.6 mm), and* C. albicans* (09.0 ± 0.3 mm) at a concentration of 300 *μ*g/disc. However, both hexane and acetone extracts failed to inhibit* E. coli* E266.

**Figure 3:**
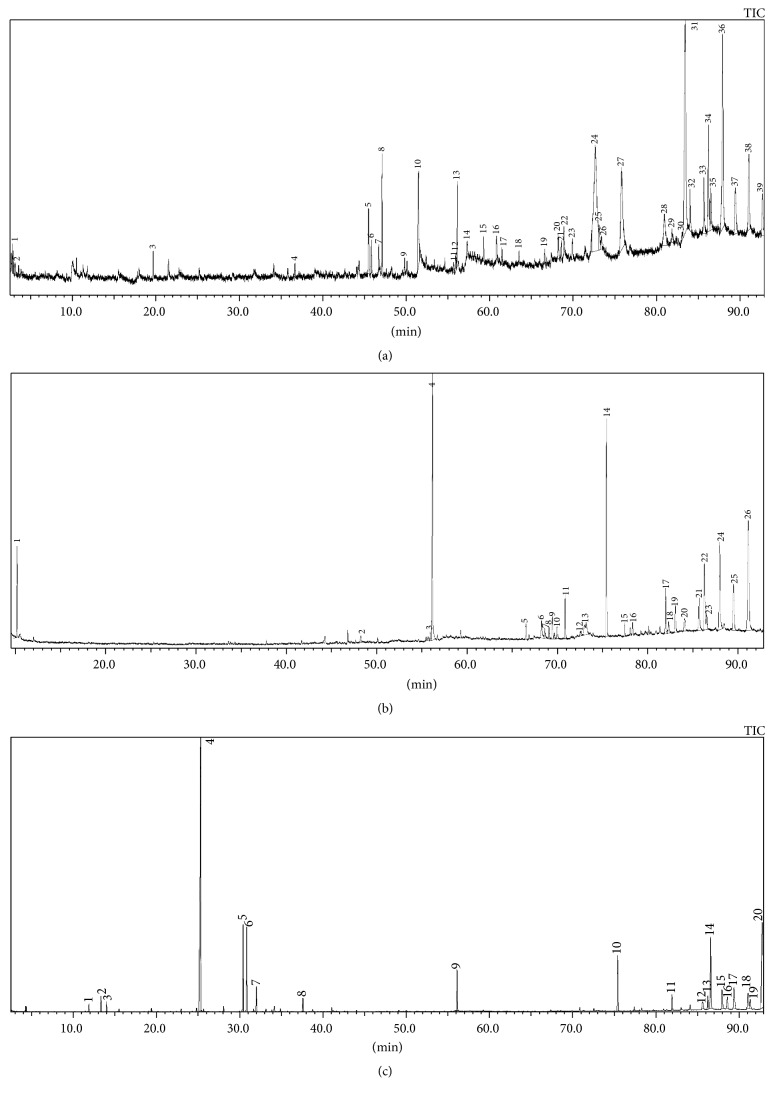
GC-MS based chemical profiling of methanol (a), acetone (b), and hexane (c) extracts from* P. amboinicus* leaves.

Further, GC-MS profiling of the extracts together revealed the occurrence of a total of 115 peaks of which about 46 chemical compounds were identified. These compounds belong to different chemical classes and most of them are reported to exhibit important biological activities.  Figure 3 shows the distinct chromatogram of* P*.* amboinicus* leaves extracted in different solvents. The identified compounds with their peak number, retention time (RT), and peak area (%) are presented in Table 4. Out of 39 peaks observed in the chromatogram of methanol extract, 19 compounds were identified with 6 major peaks. The major compounds included tetracontane (16.6%), tetrapentacontane (11.3%), pentacosane (7.8%), and n-hexadecanoic acid (4.9%). Among the 26 peaks observed in the GC-MS profile of acetone extract, only 11 compounds were detected. The major compounds identified were Phytol (12.9%), squalene (15.6%), and *β*-Amyrin (5.3%). While hexane extract profile revealed the presence of 16 detectable compounds from a total of 20 peaks, including carvacrol (37.7%), trans-caryophyllene (7.3%), squalene (4.7%), tetrapentacontane (13.7%), and tetratriacontane (8.8%) as the major components.

## 4. Discussion

Recent years have witnessed the importance of phytochemicals due to their remarkable health benefits. Plant metabolites are extracted by using various ways, such as maceration, decoction, soxhlet extraction, microwave-assisted extraction, supercritical fluid extraction, and ultrasound-assisted extraction method. As maceration technique is a simple and the easiest method, it is widely used at the preliminary research level [11]. The efficiency of extraction depends on several factors, including the nature of phytochemical constituents, the method of extraction, particle size of the sample, extraction time, temperature, pH, solute to solvent ratio, and the solvent polarity [12]. The appropriate use of solvent system is crucial in order to recover higher extract yield, polyphenols, and some other bioactive compounds from a sample [13]. Plant derived polyphenols including flavonoids possess several biological properties and thus necessitate evaluating their presence in different plant parts extracted in different organic solvents [4, 14, 15]. This study revealed the existence of a significant difference in the extract yield obtained by different solvents. Further, the highest extract yields and total phenolic content obtained in the methanolic leaf extract of* P. amboinicus *could be attributed to its high polarity [13]. Another possible reason might be due to the establishment of complexes by phenolic constituents with other biomolecules such as proteins, carbohydrates, terpenes, and inorganic compounds that can be more easily recovered from methanol in comparison to other solvents [16]. Similarly, the use of methanol as the best solvent for extraction is well documented in the literature [4, 13, 17]. On the contrary, methanol, acetone, and water are reported to be ineffective for extracting polyphenols from the seeds of grapes [18]. In the present study, the extractive yield and total phenolic content were better evidenced in the nonpolar hexane extract when compared to the polar solvent, acetone. In contrast, the hexane extract yielded much lesser total flavonoid content compared to the acetone extract. This difference in the extract yields could be attributed to the occurrence of a wide range of diverse phytochemical components in* P. amboinicus *leaves and differences in the solvent polarity. Furthermore, it is reported that the level of solubility of a phenolic compound decides the recovery percentage of a particular solvent type [4, 13]. In contrast, according to the report by Bhatt and Negi, [19], the acetone extract contained significantly higher total phenolics compared to the methanol or hexane extracts, which was endorsed for the difference in the geographic location and other environmental factors [20]. Likewise, earlier study has identified the presence of polyphenols such as caffeic acid, coumaric acid, rutin, quercetin, and gallic acid in the acetone leaf extract of* P. amboinicus* [21]. As there is a dearth of scientific details on phytochemistry of Malaysian* P. amboinicus*, this study will be certainly appreciated. In general, this study suggests the use of methanol and hexane solvents for higher recovery of extractable compound from* P. amboinicus *leaves. Because of the lower extractive yield obtained in this study, it is recommended to use other better extraction methods in* P. amboinicus*. Nevertheless, it is reported that the biological properties depend not only on the total extract yield but also on its phytochemical composition [22].

In the human body, cell damage may induce the generation of increased levels of free radicals. Various disorders including myocardial infarction, cancer, atherosclerosis, and neurogenerative disorders are mainly correlated to these free radicals [4]. Antioxidants are chemical compounds and have the ability to prevent various oxidative stress related cell damages mediated by free radicals. Many antioxidant molecules are recorded in several medicinal plants and thus will be beneficial in the treatment of several human diseases [15]. Most frequently, the DPPH and FRAP assay methods are preferred for determining antioxidant activity [9]. In this study, radical scavenging activity was observed to be concentration dependent and corroborates with the previous reports on several other plant species [4, 9, 23]. The superior radical scavenging potential of plant solvent extracts may be interrelated to the presence of various antioxidants such as polyphenolic compounds [14, 23]. Even though earlier reports have stated that* P. amboinicus *leaf extracts possess antioxidant properties [2], none of them have reported the systematic study comparing the effect of different solvents on the antioxidant potential of* P. amboinicus* species growing under tropical conditions of Malaysia. The methanol extract was found to exhibit the highest scavenging activity when compared to other solvent extracts. This could be attributed to the fact that the methanol extract possesses higher antioxidant molecules such as phenolic compounds as evidenced by our phytochemical analysis (Table 2). In contrast, Bhatt and Negi [19] reported that acetone extract of* P. amboinicus *possessed superior antioxidant activity when compared to hexane, ethyl acetate, or methanol extracts. According to Rai et al. [24], the effective antioxidant activity of* P. amboinicus* leaves was observed when extracted with the solvent, ethanol. This difference could be could be due to differences in the geographical growth conditions as stated previously by Swamy et al. [4]. As stated by the earlier reports, the free radical scavenging activity of different solvent extracts of plants mainly depends on the existence of different bioactive chemical constituents [1, 5]. Further, more polar solvents can often extract antioxidant compounds in higher quantities. Overall, high-polarity solvent (methanol) was very effective in extracting more antioxidant compounds when compared to an intermediate polar solvent, acetone, and nonpolar solvent, hexane.

Antimicrobial agents (synthetic or plant based) effectively kill a wide range of infectious microbes. However, their improper and overuse has permitted microbes to develop multidrug resistance ability which is a major threat and a bigger challenge to the medical world [25]. Hence, there is a need to investigate novel molecules to overcome antimicrobial resistance. Further, present research emphasizes on the development of plant based drugs due to the fact that synthetic drugs cause several side effects in humans. In this regard, we evaluated the antimicrobial properties of* P. amboinicus *leaf extracts and the results were very conclusive. All the tested microorganisms were effectively inhibited by all solvent extracts with the exception of a gram-negative bacteria* E. coli, *which was inhibited only by the methanolic leaf extract. MRSA strain exhibited less susceptibility to all solvent extracts when compared to other gram-positive strains.* B. subtilis* was highly susceptible to the methanol extract. Interestingly, methicillin-resistant* S. aureus* and* C. albicans* were highly susceptible to all the extracts. Similarly, Gurgel et al. [26] reported the efficacy of the hydroalcoholic leaf extracts of* P. amboinicus* from Brazil against MRSA strains while Manjamalai et al. [27] report the anticandidal activity of the methanolic leaf extract of Indian* P. amboinicus*. To support this, researchers have stated that mostly gram-negative bacteria exhibit more resistant properties against a wide range of antibiotics or chemical drugs when compared to gram-positive bacteria [19, 28]. This difference in the susceptibility of a microbe to plant extracts is also linked to differences in their cell wall and outer membrane structures [29]. MRSA cause several human health problems including skin infections, sepsis, pneumonia and other infections, and* C. albicans* is a prime causative agent of candidiasis. Thus,* P*.* amboinicus *leaf extracts may be beneficial in treating these diseases causing microbes. Microbial inhibition was found to vary with the type of solvent extracts evaluated. Likewise, previous researchers also have testified the wide-ranging antimicrobial activity of this plant [2, 19]. However, these results contradict with the findings by Bhatt and Negi [19], where the superior antimicrobial activity was shown by the acetone extract, while Jiyauddin et al. [8] report the highest antimicrobial activity in the ethanolic leaf extract. This discrepancy could be due to differences in the extraction methods and microbes tested. The highest antimicrobial activity of methanol extract against all the examined microorganisms could be due to more soluble bioactive compounds extracted in the methanol solvent. These bioactive compounds, perhaps, may inhibit microbial growth effectively by binding to their cell surface. In support of this, previous studies have stated that the antimicrobial action of plant extract is presumably related to the collective effect of phenolic compounds adsorption onto the cell membrane leading to its disruption and cell leakages and the generation of hydroperoxides by phenolic compounds [2, 30]. These results support the reports emphasizing on the curative property of* P. amboinicus *plant extracts and its essential oil [2]. However, most of the research studies have evaluated antimicrobial activities of essential oil of* P. amboinicus *and limited importance is given on different plant extracts, especially, in those plants growing under Malaysian conditions [2, 8, 20, 26, 27, 31]. Furthermore, this study establishes the importance of the solvents in recovering higher extractable compounds with potent antimicrobial action against human pathogens for the first time.

Various biological activities of* P*.* amboinicus *leaf extract can be described by investigating the chemical composition of each extract using GC-MS analysis. The literature survey reveals the occurrence of about 30 nonvolatiles from methanol, ethyl acetate, water, and chloroform extracts of* P. amboinicus *leaves, stems, and roots around the world [2, 19, 32]. However, to the best of our knowledge, there is no report of GC-MS based metabolite profiling to detect the presence of various bioactive compounds in hexane and acetone extracts of* P. amboinicus* leaves. In addition, compounds in different solvent extracts of Malaysian* P. amboinicus *plants are yet to be studied. Accordingly, GC-MS analysis was performed in the preset study and identified a total of 46 compounds from different solvent extracts. The highest number of compounds (19) was evidenced in the methanolic leaf extract followed by a hexane extract (16) and acetone extract (11). Most of these compounds are known to exhibit various pharmacological activities. In this study, the higher antioxidant and antimicrobial activities of the methanol extract could be correlated to the occurrence of more number of bioactive compounds including n-hexadecanoic acid, Phytol, and squalane. Similarly, the occurrence of these compounds is also evidenced in many medicinal plants leaves extracted with methanol [5, 33, 34]. Phytol is an important diterpene that possesses antimicrobial, antioxidant, and anticancer activities [5, 35, 36]. Hexadecanoic acid is known to exhibit strong antimicrobial and anti-inflammatory activity [5, 37]. Squalene is a triterpene that act as natural antioxidants and possess various pharmacological importance [38, 39]. Neophytadiene is a good analgesic, antipyretic, anti-inflammatory, antimicrobial, and antioxidant compound [33]. Similarly, the compounds *α* and *β*-Amyrin present in hexane and methanol extracts are known to possess antidiabetic, anti-inflammatory, antiarthritic, and anticancer activities [34]. The presence of compounds such as carvacrol, caryophyllene oxide, and stigmasterol in hexane extract is also reported with various biological activities [2, 33]. Similarly, Kunle et al. [39] also showed the occurrence of carvacrol in the hexane extract of* Lippia multiflora* leaves and showed potent antimicrobial activity [39]. Carvacrol, an isoprenyl phenol is reported as one of the strongest antimicrobial agents [40]. Moreover, it has shown to inhibit the biofilms formation by* Chromobacterium violaceum*, Typhimurium DT104,* Salmonella enterica*, and* Staphylococcus aureus* [41]. The compound, caryophyllene oxide, exhibits a wide range of antimicrobial properties [42]. Likewise, a steroid compound was also isolated from the hexane extract of* Hydnophytum formicarum* [43]. In the same study, stigmasterol was shown to possess better antimicrobial and antioxidative properties. This could be one of the reasons for the higher antimicrobial activity exhibited by hexane extract compared to acetone extract as stated earlier. However, some of the other major compounds, including tetracontane, pentacosane, tetratriacontane, tetrapentacontane, and hentriacontane, are yet to be described in detail. Nevertheless, more research efforts are required to isolate, characterize, and evaluate these compounds from* P. amboinicus *leaves to validate their various pharmacological importance.

## 5. Conclusion

In this study, the presence of various soluble bioactive compounds in* P. amboinicus *leaf extracts greatly contributed to antioxidant and antimicrobial activities. Phytochemical composition and yield of the extract varied depending on the solvent types used for the extraction. In the methanolic extract, more soluble phytocompounds were noticed. The results clearly support the use of* P. amboinicus *in traditional medicinal practices to treat various diseases. Thus, this plant can serve as a new natural source for obtaining many therapeutically valued metabolites against various diseases.

## Figures and Tables

**Table 1:** Dry weight and total yield of different solvent extracts of *P. amboinicus *leaves.

Solvent extracts	Weight of the extract^*∗*^ (mg ± SD)	Yield (%)
Methanol	351.53 ± 4.31^a^	4.0
Acetone	111.6 ± 3.01^c^	1.0
Hexane	131.56 ± 2.69^b^	1.32

^*∗*^Each value is expressed as mean ± standard deviation (SD) (*n* = 3).

Values in the column followed by a different letter superscript are significantly different (*p* < 0.05).

**Table 2:** Total phenolics and flavonoids content of different solvent extracts of *P. amboinicus *leaves.

Solvent extracts	Total phenolic content^*∗*^ (mg GAE/g) ± SD	Total flavonoid content^*∗*^ (mg RE/g) ± SD
Methanol	94.37 ± 1.24^a^	26.90 ± 1.35^a^
Acetone	63.20 ± 1.22^c^	21.27 ± 0.80^b^
Hexane	75.39 ± 1.07^b^	16.46 ± 0.99^c^

^*∗*^Each value is expressed as mean ± standard deviation (SD) (*n* = 3). Values in the column followed by a different letter superscript are significantly different (*p* < 0.05) and values having the same letters are not statistically significant (*p* < 0.05). GAE: gallic acid equivalent and RE: rutin equivalent.

**Table 3:** Antibacterial activity of different solvent extracts of *P. amboinicus *at different concentrations.

Solvent extracts (*μ*g/disc)	Zone of inhibition^*∗*^ (mm)				
	*Bacillus subtilis* *B29*	*Staphylococcus aureus* (*MRSA*)^*∗∗*^	*Pseudomonas aeruginosa* *ATCC 15442*	*Escherichia coli* *E266*	*Candida albicans* *90028*
Hexane					
100	06.1 ± 0.1	08.0 ± 0.3	03.9 ± 0.3	—	06.1 ± 0.2
200	06.9 ± 0.4	09.2 ± 0.2	06.0 ± 0.2	—	06.9 ± 0.4
300	08.2 ± 0.1	10.0 ± 0.3	07.0 ± 0.2	—	07.9 ± 0.3
Methanol					
100	08.1 ± 0.2	07.1 ± 0.5	06.1 ± 0.2	06.0 ± 0.2	07.1 ± 0.2
200	08.9 ± 0.1	08.0 ± 0.5	06.2 ± 0.3	07.4 ± 0.5	08.3 ± 0.3
300	10.2 ± 0.5	09.2 ± 0.3	07.2 ± 0.2	08.7 ± 0.6	09.0 ± 0.3
Acetone					
100	01.9 ± 0.1	05.2 ± 0.3	02.2 ± 0.2	—	05.6 ± 0.3
200	02.9 ± 0.2	06.1 ± 0.4	03.2 ± 0.3	—	06.0 ± 0.2
300	03.0 ± 0.1	08.4 ± 0.5	03.9 ± 0.2	—	06.8 ± 0.3

^*∗*^The experiment included DMSO (20 *μ*L) as negative control while streptomycin (100 mg/mL) for bacteria and nystatin (100 mg/mL) for yeast served as positive control. Each value represents the mean ± standard deviation (SD) of 3 replicates per treatment in 3 repeated experiments.

“—” represents no activity observed.

^*∗∗*^MR represents Methicillin resistant.

**Table 4:** GC-MS profile of different solvent extracts of *P. amboinicus *leaves.

S. number	Name of the compound	Peak number	R. time	Peak area (%)
*Methanol extract*				

(1)	Butanoic acid, methyl ester	2	03.14	00.35
(2)	5-Methoxypyrrolidin-2-one	3	19.69	00.72
(3)	(−)-Loliolide	5	45.52	02.15
(4)	Neophytadiene	7	46.75	00.81
(5)	2-Pentadecanone, 6,10,14-trimethyl-	8	47.11	03.01
(6)	1-Decanol, 2-hexyl-	9	49.84	00.49
(7)	n-Hexadecanoic acid	10	51.46	04.97
(8)	Methyl (Z)-5,11,14,17-eicosatetraenoate	12	55.93	00.50
(9)	Phytol	13	56.13	03.30
(10)	Phytol, acetate	15	59.30	00.61
(11)	Octanoic acid, 2-dimethylaminoethyl ester	17	61.48	00.39
(12)	Carbonic acid, 2-dimethylaminoethyl isobutyl ester	19	66.60	00.51
(13)	Di-n-octyl phthalate	21	68.59	00.66
(14)	Tetrapentacontane	24	72.63	11.32
(15)	Pentacosane	27	75.78	07.88
(16)	Triacontane	28	80.93	02.60
(17)	Tetracontane	31	83.42	16.67
(18)	Squalane	35	86.54	01.73
(19)	Methyl commate A	38	91.08	01.96

*Acetone extract*				

(20)	9,12-Octadecadienoyl chloride, (Z,Z)-	3	55.93	00.63
(21)	Phytol	4	56.15	12.95
(22)	Glycerol 1-palmitate	6	68.25	01.73
(23)	Di-n-octyl phthalate	7	68.62	00.47
(24)	(2,3-Diphenylcyclopropyl) methyl phenyl sulfoxide	8	69.07	00.93
(25)	Nerolidol propionate	11	70.88	02.76
(26)	Squalene	14	75.454	15.64
(27)	Nonacosane	15	77.45	01.01
(28)	2,2,4-Trimethyl-3-(3,8,12,16-tetramethyl-heptadeca-3,7,11,15-tetraenyl)-cyclohexanol	16	78.34	00.86
(29)	Hentriacontane	17	81.98	03.37
(30)	*β*-Amyrin	25	89.50	05.34

*Hexane extract*				

(31)	Benzene, 1-methyl-3-(1-methylethyl)-	1	11.91	00.49
(32)	*γ*-Terpinene	2	13.37	01.03
(33)	trans Sabinene hydrate	3	14.04	00.66
(34)	Carvacrol	4	25.32	37.73
(35)	trans-caryophyllene	5	30.40	07.37
(36)	*α*-Humulene	7	32.03	02.15
(37)	Caryophyllene oxide	8	37.59	01.00
(38)	Phytol	9	56.13	03.19
(39)	Squalene	10	75.43	04.75
(40)	Pentacosane	11	81.95	00.86
(41)	Stigmasterol	13	86.25	01.29
(42)	Tetratriacontane	14	86.57	08.80
(43)	Hexatriacontane	16	88.57	01.02
(44)	*α*-Amyrin	18	91.07	01.53
(45)	Hexatriacontane	19	91.33	01.47
(46)	Tetrapentacontane	20	92.76	13.77
